# The lethal and sterile doses of gamma radiation on the museums pest, varied carpet beetle, *Anthrenus verbasci* (Coleoptera: Dermestidae)

**DOI:** 10.1038/s41598-023-43739-x

**Published:** 2023-10-09

**Authors:** Ali Hamza, Nagwan Zahran, Nagwa El shafeay

**Affiliations:** 1 Department of Natural Products Research, National Center for Radiation Research and Technology (NCRRT), Egyptian Atomic Energy Authority (EAEA), Cairo, Egypt; 2 Center of Researches and Conservation of Antiquities, Ministry of Antiquities, Cairo, Egypt

**Keywords:** Biological techniques, Zoology

## Abstract

Museums preserve historical cultural artifacts and serve as an essential resource for current and future generations seeking first-hand knowledge about the diversity of life on Earth. However, significant changes in climate from temperature and humidity cause serious biotic degradation. Despite ongoing insect control treatments, insect pests are still a major problem for museums due to the lack of suitable and unsafe environments that are provided for the storage and display of the collection. The varied carpet beetle, *Anthrenus verbasci* (Coleoptera: Dermestidae) is one of the major stored product pests whose larvae cause serious damage to household items and museum specimens. Therefore, this research aims to study the effect of gamma radiation on the larval, pupal and adult stages. The effects of gamma radiation have been studied on 3rd instar larvae (100, 200, 300, 400 and 500 Gy). The results showed that mortality in the larval stage significantly increased with increasing gamma radiation dose, which was reflected in the eclosion of the adult stage. The exposure of one-day-old pupae to 200, 400, 600, 800 and 1000 Gy of gamma radiation showed that the higher the dose, the lower the percentage of adult emergence. Additionally, there was a significant increase in the percentage of mortality in *A. verbasci* adults with increasing radiation doses when the newly emerged adults were irradiated with 200, 400, 600, 800 and 1000 Gy. The LD_50_ and LD_90_ of gamma radiation doses on larvae, pupae and adults were calculated, and the malformations in all stages were photographed. The fecundity and fertility of *A. verbasci* adults that were exposed to radiation as one-day-old pupae decreased gradually with increasing doses of gamma radiation and reached 100% sterility when exposed to a dose of 150 Gy. Among all the treatments, the sterile dose (150 Gy) or lethal dose (1000 Gy) showed superior performance over other treatments and was adjudged as the best treatments, which prevented the subsequent development and complete mortality of the pest.

## Introduction

Today, museums are important institutions that affect not only culture but also tourism, the economy, and national identity. Preserving art and other artifacts of cultural heritage in the best possible condition for future generations is a major challenge. Insects are considered among the most impacting biodeteriogens for organic items in museums and other indoor settings^1^. There are approximately 70 exceedingly harmful species that use artifacts as sources of nutrition and shelter and as places to lay eggs^1^. A diverse number of approximately 1500 species globally distributed belong to Dermestidae (Insecta: Coleoptera)^2^. Some carpet beetle species feed on dried preserved museum specimens, while others feed on books, rye, cereals, red pepper, flour, mill powder, etc. Damage is always done by larvae, while in some species; adults were found feeding on flower pollens, rugs, tables, bookshelves, clothing, etc.^3^.

The varied carpet beetle, *Anthrenus verbasci* (Linnaeus, 1767), infests museum collections such as carpets, woolen items, silks, skins, furs, feathers, hair, horn, cereals, red pepper, fishmeal, or any processed animal or plant food. They live indoors and outdoors, anywhere that they can find a lot of food. Varied carpet beetles are from Europe, Asia, and North Africa, but came to North America around 1850. Outdoor adult varied carpet beetles can be found on flowering plants, wasp nests, nests of birds and under the siding of homes. Indoors they are found in stored food materials, plant materials (dried fruit and nuts), and animal materials (wool, fur, and skins)^4–7^. The adult carpet beetle appears during May and feeds only on nectar and pollen of white flowers of *Chrysanthemum* spp.*, Erigeron annuus,* etc., but lays its eggs in accumulated animal stuff in buildings; they are also common inhabitants of bird nests^8,9^. Adults' sizes range in length from 2 to 3.5 mm, and they have a distinctive pattern of white, brownish, and yellowish scales on their backs, as well as fine, long, grayish-yellow scales below. The mature larva measures 4 to 5 mm in length and has a pattern of transverse light- and dark-brown stripes. Additionally, they are commonly known as woolly bears and if it is suddenly alarmed, the larva erects the three dense tufts of bristles and hair located on each side of the rear end of the body. *Anthrenus verbasci* has an unusual life cycle for an insect, developing from larvae to adults in 1–3 years, depending on the environmental conditions. The female lays about 40 eggs. There are usually 7–8 larval instars, but the number may vary from 5 to 16. The numbers of days for the various stages are as follows: egg, 17–18; larva, 222–323; and pupa, 10–30. The period from egg to adult is 249–354 days, and the adult may live another 14 to 44 days^10^. The larvae of the carpet beetle are voracious feeders and can cause significant damage to wool, eventually in dry dead insects specimens in the museum collections^11^. The appearance of the infestation of the larvae of these beetles on clothes and carpets can be recognized by small, clean holes that are not accompanied by threads of silk fabric (as is usually the case with clothing moth infestations), but have a powdery appearance like dust. Additionally, the transparent skins of the larvae are also a conspicuous sign of infestation^12^.

Among the various methods for insect control, the use of insecticides is the most widely applied and this poses several problems, such as resistance in insects to chemicals, environmental pollution and hazards to non-target biota^13,14^. *Anthrenus verbasci* adults are relatively easy to control using residual insecticide treatments^15^, although the treatment must coincide with adult activity and egg laying. The larvae, however, are more difficult to control as they often remain hidden within the food source and take a relatively long time to succumb to insecticide treatment, even after picking up a lethal dose^16^. Insecticidal sprays, dusts or vapors are commonly applied for control of museum pests. Such application may be harmful to museum articles^17^. To overcome some of the negative effects of pesticides, the use of gamma radiation is an alternative option^18^. All stages of insect pests can be killed with different nonchemical methods, and these methods are preferred in museums, libraries and historic buildings, as they do not damage objects and do not harm the environment or the health of museum staff IAEA^19^. Some alternative methods have shown efficiency in controlling pests, such as ionizing radiation^20^. Due to the need for better conservation and control of insects with modern technology, high efficiency, low cost and the absence of side effects, gamma radiation is considered the most viable solution. This process inhibits reproduction or even causes the death of infesting insects^21^. However, for such control, it is of prime importance to know the lethal doses of ionizing radiation for the different stages of the life cycle of the pest, as radiosensitivity varies according to several factors, including the stage of development Arthur et al.^22,23^. IAEA^19^ recommended that the treatment dose of gamma radiation in disinfestations of cultural heritage artifacts in the case of an insect attack should be at least 0.5 kGy, but can be up to 2 kGy a dose also effective for the eradication of insect eggs.

Radiation is a common and reliable method for inducing procreative sterility that keeps the released insects' level of aggression at low cost^24^.

The most radiosensitive cells are those with a high mitotic rate, with a long mitotic future and which are of a primitive type. These generalizations, with some exceptions, have become known as the Law of Bergonie and Tribondeau^25^. In this regard, germ cells are the most radiosensitive, and show different killing and sterilization susceptibility according to their development stage. It is generally accepted that chromosomal damage (structural and numerical anomalies) is the cause of dominant lethal mutations. Dominant lethal mutations occurring in a germ cell do not cause dysfunction of the gamete, but are lethal to the fertilized egg or developing embryo^26^. The earlier stages of spermatogenesis (spermatocytes and spermatogonia) are generally more radiosensitive than later stages (spermatids and spermatozoa)^27^. Germ-cell sensitivity in female insects is, however, complicated by the presence of nurse cells that are most susceptible to injury during mitosis^28^.

The aim of this research is to determine the lethal doses of different physiological stages of the varied carpet beetle, *A. verbasci* in laboratory conditions and the sterilizing dose of this pest insect irradiated at pupal stage by gamma radiation to control it in stores and museums.

## Materials and methods

### Insects rearing

A laboratory population of the varied carpet beetle, *Anthrenus verbasci* was supplied by the Center of Researches and Conservation of Antiquities, Ministry of Antiquities. Cairo, Egypt. Efficient mass rearing for *A. verbasci* at different physiological stages was carried out to provide experimental work at the laboratory of entomology at the National Center for Radiation Research and Technology (NCRRT). Adults of *A*. *verbasci* were kept in plastic boxes (6 cm × 4 cm × 2 cm) and maintained on a diet of honey and milk powder (1:1) under conditions of 16 h light: 8 h dark at 30 ± 1 °C. Pieces of wool were provided as oviposition substrates, and every day, the wool onto which eggs had been laid was transferred to glass jars (350 ml) covered with muslin and secured by rubber bands, then supplied with hen feathers as larval food. The hatching of larvae was examined every day and the number of larvae in a jar was restricted to 20 or fewer in order to maintain a low density. Within a week after hatching, the glass jars were transferred either to LD 12 h light: 12 h dark at 30 ± 1 °C. The pupation of larvae was recorded each week with removing the pupae daily to prevent cannibalism.

## Irradiation technique

The irradiation source used in the present study on Carpet Beetle, *A. verbasci* was a 220 Gamma Cell Irradiation Unit (CO_60_ source) Located at NCRRT. Experiments started when the dose rate was 1.003KGy/h.

## Experimental techniques

### Larval, pupal and adults experiments to determine the lethal dose of gamma radiation

The previous glass jars which containing the adults of *A. verbasci* incubated until females started to oviposit their eggs on the pieces of wool. After 24 h the pieces of wool that contain one-day-old eggs were removed from the jars and put in other jars containing hen feathers, and incubated at 30 ± 1 °C. After the eggs hatch and the larvae emerge, the exuvia are followed up and removed until the pest reached the 3rd instar larvae, then they were irradiated with five dose levels ranging from 100 to 500 Gy increments.

Pupae (one-day-old) and newly emerged adults were transferred to plastic boxes (6 cm × 4 cm × 2 cm) and then exposed to five dose levels that ranged from 200 to 1000 Gy increments. After the irradiation treatment, both pupae and adults transfered into glass jars containing hen feathers. The numbers of dead larvae, pupae and adults were counted daily for three weeks. Records were kept for the mean number of dead larvae, % reduction, % mortality and adult emergence, to determine the lethal dose for each stage. At the same time, a control population was given the same treatment conditions except irradiation. Three experimental replicates for each dose were carried out and each replicate contained ten numbers of each insect stage.

### Assessment of *A. verbasci* adults sterility

To evaluate the sterilization effect of radiation for both sexes of *A. verbasci*, mating was performed between newly emerged adults (I ♂ × I ♀) resulting from one-day-old irradiated pupae (50, 100 and 150 Gy) and compared to the control (N ♂ × N ♀). One pair of irradiated newly emerged male and virgin female were combined in a suitable oviposition cage (a Petri dish containing drop of honey and milk powder with pieces of wool) to mate and oviposit. The total numbers of eggs were counted daily under a binocular microscope. Five replicate experiments were carried out for each dose. Egg production, hatchability and % sterility were calculated.

### Statistical analysis

Data collected from the above experiments were subjected to statistical analysis, where values were represented as their percentages or mean ± SE. To compare differences in means, one-way analysis of variance (ANOVA) was performed using the statistical software program CoStat (1995). The percentage of reduction in different stages (larvae, pupae and adult) was calculated by the formula according to Abbott^29^:

$$\% {\text{ Reduction }} = \frac{ C - T}{C} \times 100$$ × 100.

where C = mean number of insect stages in the control. T = mean number of insect stages in the treatment.

Percentage sterility was calculated according to Chamberlain's formula as mentioned by Guirguis^30^:

$$ \% {\text{ Sterility }} = {1}00 \, {-} \left( {\frac{ a \times b}{{A \times B}} \times 100} \right) $$

where a = number of eggs/female in treatment, b = % hatch in treatment; A = number of eggs/female in control, B = % hatch in control.

## Results

### Effect of gamma radiation on different stages of *Anthrenus verbasci*

To evaluate the lethal dose for different stages of *Anthrenus verbasci*, 3^rd^ instar larvae were irradiated with 100, 200, 300, 400 and 500 Gy, and one-day-old pupae and newly emerged adults were exposed to 200, 400, 600,800 and 1000 Gy.

### Effects of gamma radiation on *A. verbasci* treated as larvae

Table 1 illustrates the impact of gamma radiation on 3^rd^ instar larvae of the varied carpet beetle, *A. verbasci*. Mortality in the larval stage significantly increased with increasing gamma radiation dose, which reflected on the eclosion of the varied carpet beetle adults. The total mean numbers of larval mortality were 4.3, 6.0, 7.7, 9.0 and 10.0 larvae when irradiated with 100, 200, 300, 400 and 500 Gy, respectively, compared to 0.0 in the control. The highest numbers of larval mortality occurred at the 1st week of inspection at all doses and were 1.7, 2.7, 4.7, 7.0 and 10.0 larvae, respectively, compared to 0.0 in the control. There was a significant positive correlation between the doses of gamma radiation and the reduction percentage, which was the highest possible (90.0 and 100.0%) at doses of 400 and 500 Gy, respectively.

**Table 1:** Mean mortality numbers of *A. verbasci* larvae and the reduction percentages.

Dose (Gy)	Mean number of larval mortality after			Total	% Reduction
	1st week	2nd week	3rd week		
0	0.0** ± **0.0^a^	0.0** ± **0.0^a^	0.0** ± **0.0^a^	0.0** ± **0.0^a^	0.0^a^
100	1.7** ± **0.3^b^	1.7** ± **0.3^b^	1.0** ± **0.0^b^	4.3 ± 2.1^b^	43.3^b^
200	2.7** ± **0.3^b^	2.3** ± **0.3^b^	1.0** ± **0.0^b^	6.0 ± 2.4^c^	60.0^c^
300	4.7** ± **0.3^c^	2.0** ± **0.5^b^	1.0** ± **0.0^b^	7.7 ± 2.9^d^	76.6^d^
400	7.0** ± **0.5^d^	1.7** ± **0.3^b^	0.3** ± **0.3^a^	9.0 ± 1.4^de^	90.0^de^
500	10.0** ± **0.0^e^	0.0** ± **0.0^a^	0.0** ± **0.0^a^	10.0** ± **0.0^e^	100.0^e^
F value	122.4	9.4	13.6	51.2	
L.S.D. 5%	1.1	1.1	0.4	1.6	

*Common letter following the mean indicates no significant difference between means in a column.

**Figure 1:**
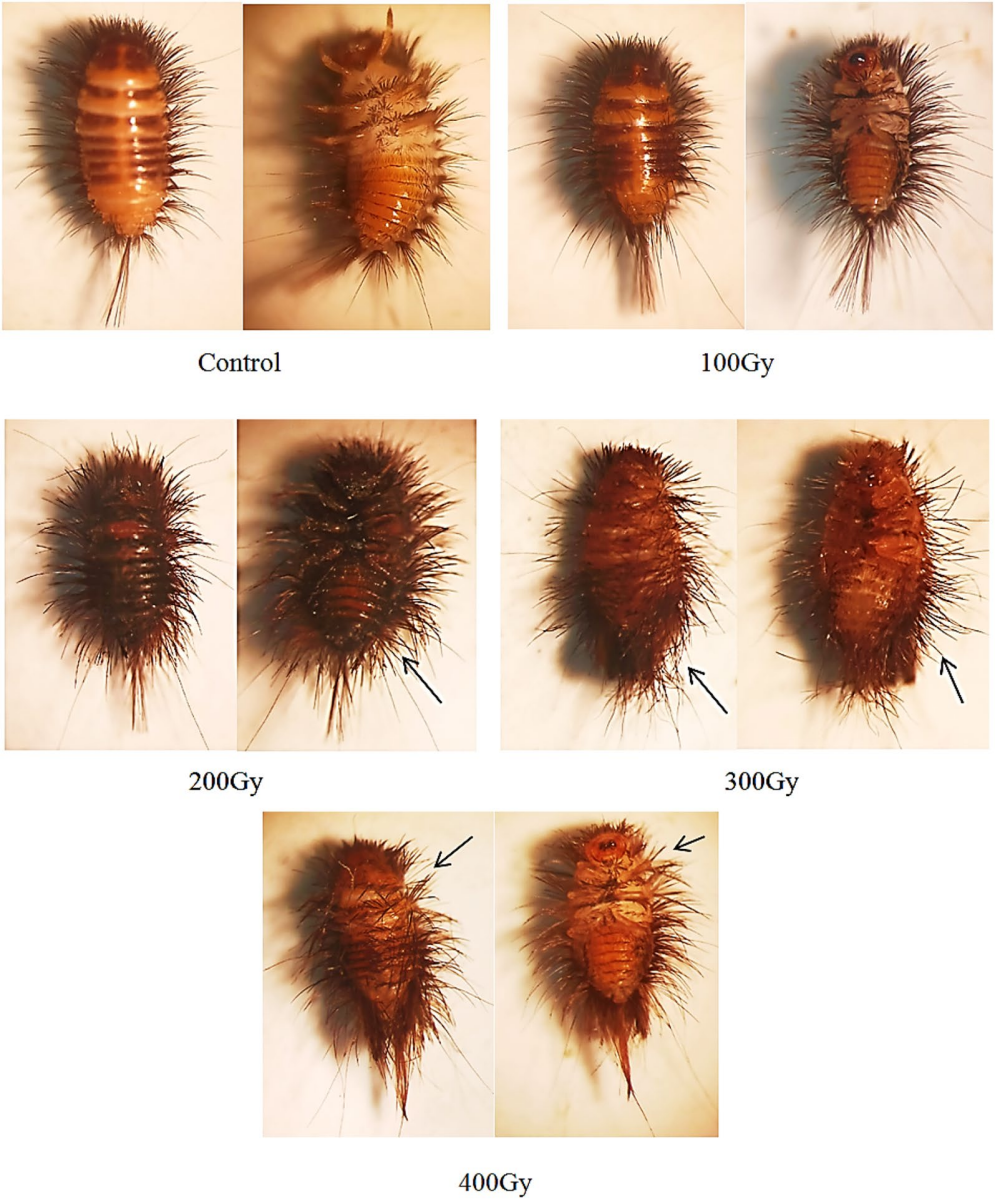
Effect of different gamma radiation doses on 3rd instar larvae of *Anthrenus verbasci.* The arrows indicate the disorganization of hairs covering the body of the larvae, broken and decreased in density.

When compared to unirradiated larvae, the exposure of *Anthrenus verbasci* third instar larvae to 100 Gy of gamma radiation had no morphological impact. As the radiation dose was increased, the various malformational characteristics gradually worsened. Figure 1 shows that the deformation developed noticeably as the overall shape of the larva became aberrant as the radiation dose increased from 200 to 400 Gy. It was observed that the hairs scattered across the wall body had uneven and diverse shapes. Additionally, compared to the control, it exhibits broken and reduced in density.

### Effects of gamma radiation on *A. verbasci* treated as pupae

The data in Table 2 clearly demonstrate that there was a significant relationship between the dose levels of gamma radiation. The exposure of 1-day-old pupae to different levels of gamma radiation (200 up to 1000 Gy) showed that the higher the dose was, the lower the percentage of adult emergence, which reached 0% at a dose of 1000 Gy. It was noted that the irradiation of pupae led to a delay in the emergence of adult insects, especially at doses of 600 and 800, as they did not emerge except in the third week, compared to nonirradiated pupae, which emerged in the first week. After 3 weeks, the reduction percentages of emerged adults from irradiated pupae with 200, 400, 600, 800 and 1000 Gy were 39.7, 56.7, 73.3, 93.3 and 100%, respectively (Table 2).

**Table 2:** Mean number of emerged adults and reduction percentage of *A. verbasci* irradiated as pupae.

Dose (Gy)	Mean number of emerged adults after			Total	% Reduction
	1st week	2nd week	3rd week		
0	7.0 ± ^a^	3.0 ± ^ab^	0.0 ± 0.0^a^	10.0 ± 0.0	0.0^a^
200	2.3 ± ^b^	4.0 ± ^a^	0.0 ± 0.0^a^	6.3 ± 1.4	39.7^a^
400	0.3 ± ^c^	2.3 ± ^b^	1.7 ± ^b^	4.3 ± 0.8	56.7^b^
600	0.0 ± 0.0^c^	0.0 ± 0.0^c^	2.7 ± ^c^	2.7 ± 0.8	73.3^c^
800	0.0 ± 0.0^c^	0.0 ± 0.0^c^	0.7 ± ^a^	0.7 ± 1.6	93.3^d^
1000	0.0 ± 0.0^c^	0.0 ± 0.0^c^	0.0 ± 0.0^a^	0.0 ± 0.0	100.0^e^
F value	84.2	24.6	22.2	190.2	
L.S.D. 5%	9.4	11.1	7.3	8.4	

*Common letter following the mean indicates no significant difference between means in a column.

**Figure 2:**
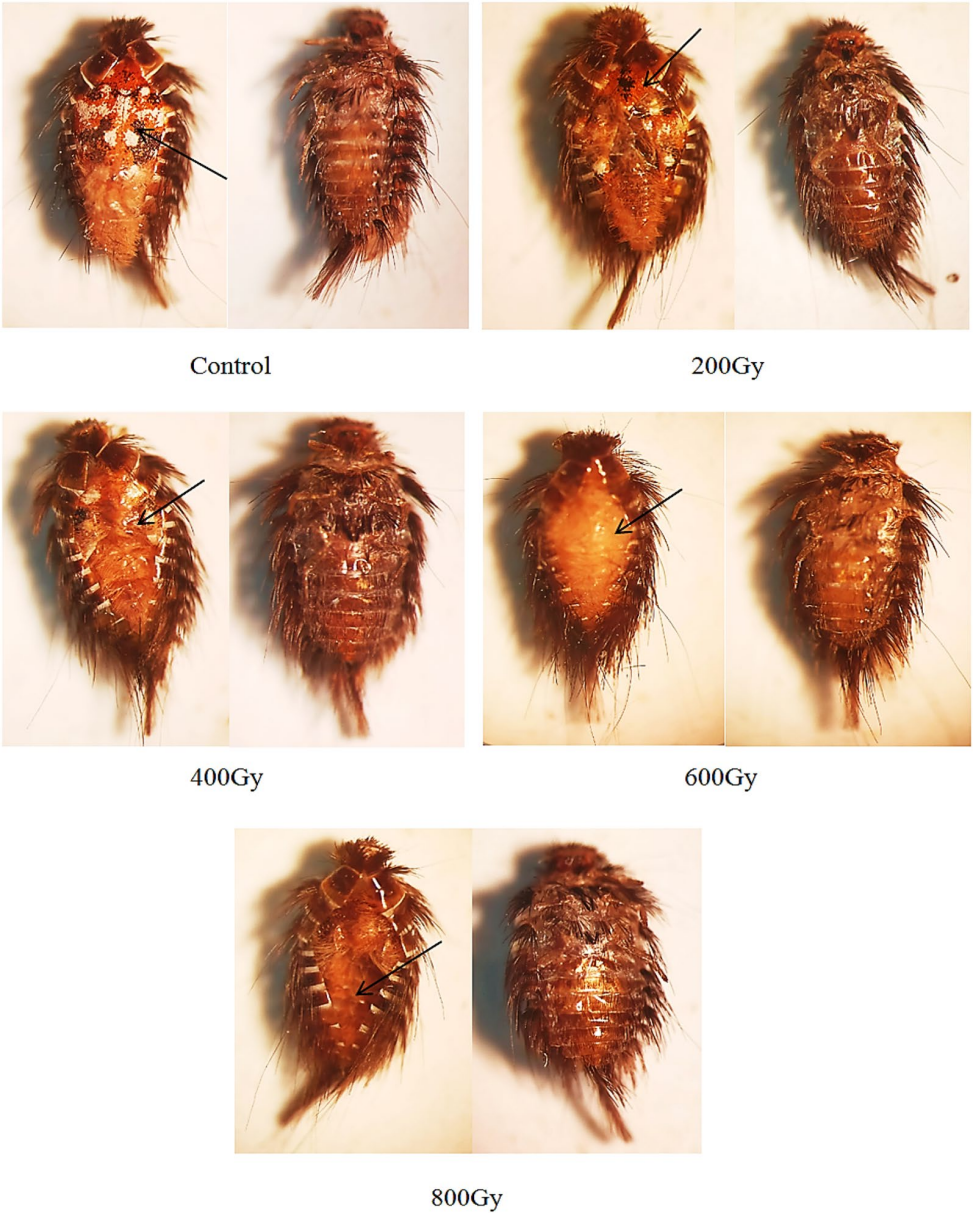
Effect of different gamma radiation doses on pupae of *Anthrenus verbasci.* The arrows indicate that the elytron cuticle of the adult insect was incomplete (200 and 400 Gy). Cuticle formation did not occur (600 Gy). The pupae decomposed completely from the inside (800 Gy).

In *A. verbasci* pupae exposed to all levels of radiation, no obvious deformities emerged; nevertheless, as the radiation dose incresing, the rate of adult emergence decreased, and the reason for this was clear from the microscopic examination of the pupae. Figure 2 shows that when pupae were subjected to 200 and 400 Gy, the adult insect's elytra cuticle was not complete, which prevented it from leaving its puparium and ultimately caused its death. Additionally, the 600 Gy dose did not result in cuticle formation. At 800 Gy of gamma radiation, the pupae fully disintegrated from the inside and transformed into an empty void, and adults did not form.

### Effects of gamma radiation on *A. verbasci* treated as adults

The data in Table 3 show that there was a significant increase in the percentage of mortality in *A. verbasci* adults with increasing radiation doses. The reduction percentage increased gradually with increasing radiation dose to 13.3, 33.3, 56.7, 76.7 and 100% at dose levels of 200, 400, 600, 800 and1000 Gy, respectively. The adult stage was more tolerant than both the larval and pupal stages, as there was no death during the first week when irradiating the whole adults with all doses except for the high doses of 800 and 1000 Gy, and the mean numbers were 0.7 and 1.7 insects, respectively. After three weeks, the reduction reached 100% for adults irradiated with the highest dose (1000 Gy).

**Table 3:** Mean number of dead adults and reduction percentage of *A. verbasci* adults.

Dose (Gy)	Mean number of adults mortality after			Total	% Reduction
	1st week	2nd week	3rd week		
0	0.0 ± 0.0^a^	0.0 ± 0.0^a^	0.0 ± 0.0^a^	0.0 ± 0.0	0.0^a^
200	0.0 ± 0.0^a^	0.3 ± 0.3^a^	1.0 ± 0.5^a^	1.3 ± 1.6	13.3^a^
400	0.0 ± 0.0^a^	0.3 ± 0.3^a^	3.0 ± 0.5^b^	3.3 ± 1.6	33.3^b^
600	0.0 ± 0.0^a^	1.7 ± 0.3^b^	4.0 ± 0.3^b^	5.7 ± 1.6	56.7^c^
800	0.7 ± 0.3^b^	3.0 ± 0.3^b^	4.7 ± 0.3^b^	7.7 ± 2.2	76.7^d^
1000	1.7 ± 0.3^c^	4.0 ± 0.5^c^	4.3 ± 0.7^b^	10 ± 0.0	100.0^e^
F value	12.5	18.4	11.7	41.6	
L.S.D. 5%	0.6	1.1	1.7	1.8	

*Common letter following the mean indicates no significant difference between means in a column.

**Figure 3:**
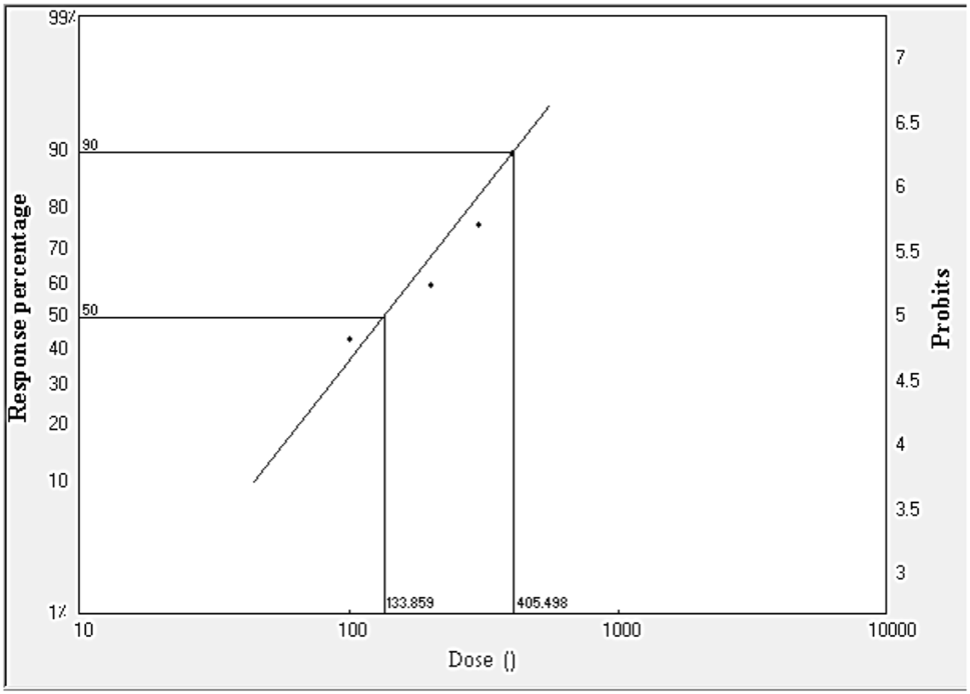
LD_50_ and LD_90_ of gamma radiation doses for 3rd instar larvae of *A. verbasci.*

**Figure 4:**
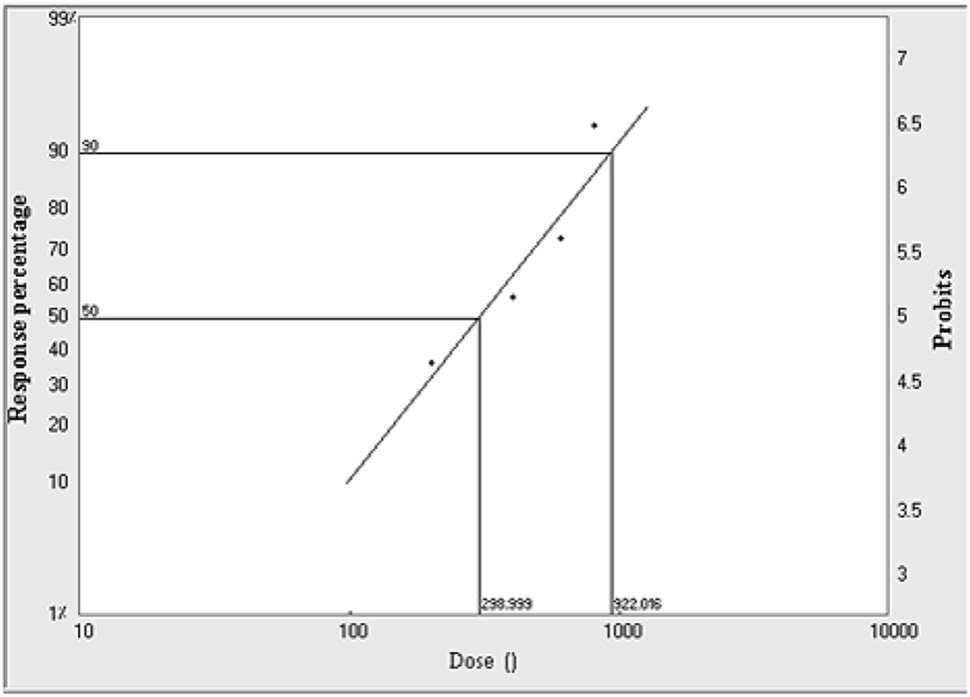
LD_50_ and LD_90_ of gamma radiation doses for 1-day–old pupae of *A. verbasci.*

**Figure 5:**
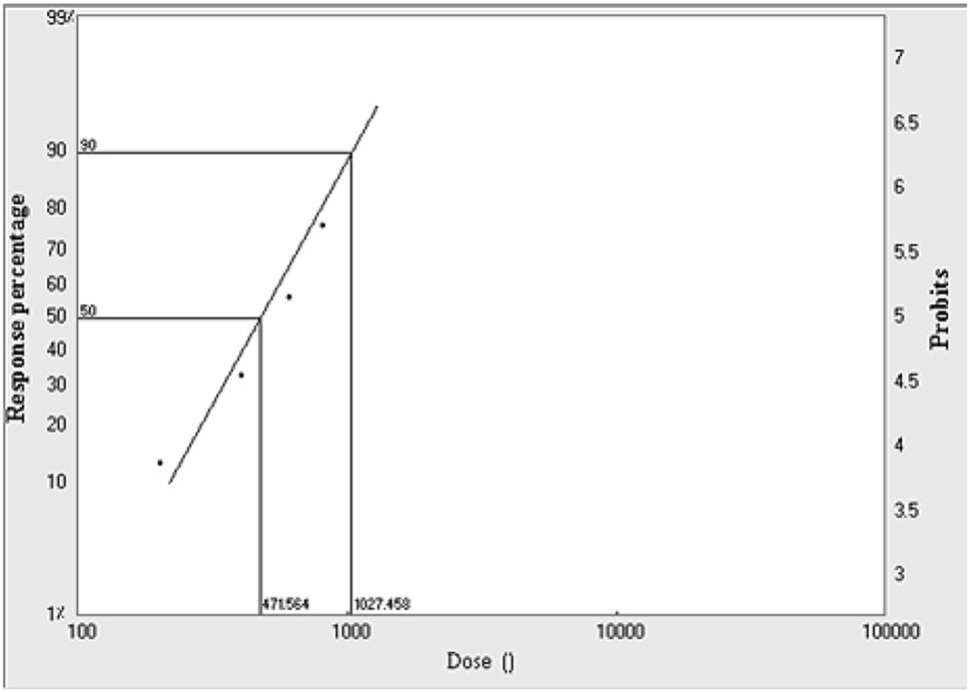
LD_50_ and LD_90_ of gamma radiation doses for newly emerged adults of *A. verbasci.*

From Table 4 and Figs. 3, 4, 5, LD_50_ and LD_90_ values were calculated for larvae, pupae and adults, which were found to be 133.9, 298.9 and 471.6 Gy, respectively for the LD_50_ and 405.5, 922.0 and 1027.5 Gy, respectively, for the LD_90_. From these values, it is clear that adults were more tolerant of radiation than pupae and larvae.

**Table 4:** LD_50_ and LD_90_ of gamma radiation doses for larvae, pupae and adults of *A. verbasci*.

Stage	LD_50_^a^ (Gy)	95% confidence limits (μl/L)		LD_90_^b^ (Gy)	95% confidence limits (Gy)		Slope^c^ ± SE	(χ^2^)^d^	*P*^*e*^
Larvae	133.9	91.6	168.2	405.5	319.9	608.8	2.7 ± 0.5	3.6	0.3
Pupae	298.9	209.6	373.1	922.0	704.1	1530.6	2.6 ± 0.5	2.7	0.4
Adult	471.6	399.6	543.3	1027.5	847.1	1402.1	3.8 ± 0.6	6.4	0.1

^a^The dose causing 50% mortality.

^b^The dose causing 90% mortality.

^c^Slope of the dose -mortality regression line ± standard error.

^d^Chi square value.

^e^Probability value.

### Effect of gamma radiation on fecundity and fertility of *A. verbasci* irradiated as 1 day old pupae

As shown in Table 5, there were statistically significant differences between the average number of eggs laid by a single irradiated female when mating with an irradiated male (I♂ × I ♀) and those laid by a normal female with a normal male (N♂ × N ♀). The fecundity (mean number of eggs/female) gradually decreased to 54.8, 46.2 and 26.6 eggs at dose levels of 50, 100 and 150 Gy, respectively, compared to 103.8 eggs in the control (normal female inseminated with a normal male). The results also show a highly significant reduction in fertility (percentage of egg hatch) throughout all dose levels when irradiated females were allowed to mate with irradiated males; the percentage of egg hatchability was 63.5, 22.1 and 0% at doses of 50, 100 and 150 Gy respectively, compared to 90.4% in the control. Additionally, the reduction percentages were 47.2, 55.5 and 74.3% at dose levels of 50, 100 and 150 Gy respectively. When sterility percentages were calculated, we found that they reached 100% (complete sterility) at a dose of 150 Gy.

**Table 5:** Effect of gamma-irradiation doses on fecundity, fertility and sterility of *A. verbasci* irradiated as one-day-old pupae.

Dose (Gy)	Mean N. of eggs/female	% Egg hatch	% Reduction	% Sterility
0	103.8 ± 31.6^a^	90.4 ± 30.8^a^	0.0	0.0
50	54.8 ± 23.3^b^	63.5 ± 14.0^b^	47.2	62.9
100	46.2 ± 15.5^b^	22.1 ± 6.5^c^	55.5	89.1
150	26.6 ± 11.2^c^	0.0 ± 0.0^c^	74.3	100.0
F value	43.9	119.7		
L.S.D. 5%	14.6	11.5		

*Common letter following the mean indicates no significant difference between means in a column.

## Discussion

A significant portion of museum collections worldwide are made up of items made of organic materials such as leather and parchment, which are quickly attacked by insects such as dermestid beetles, tenebrionides, silver fish, cockroaches, and other microorganisms^31^. Pesticides may react adversely with museum materials and taxidermies, so their use is frequently very controlled.^32,33^. The effects of gamma irradiation on different Dermestidae insects on their biological parameters were reported earlier, for example but not limited to Tilton et al.^34^ on *Trogoderma glabrum* (Herbst, 1783) and the black carpet beetle, *Attagenus piceus* (Olivier, 1790); Mansour^35^ on *Trogoderma granarium* (Everts, 1899) and Yasmin et al.^36^ on *Dermestes frischii* (Kugelann, 1792). This is the first study on the effect of gamma radiation on the museum pest, the varied carpet beetle, *A. verbasci*.

The results of our experiment indicate that the larvae were the most susceptible stages to radiation and that the adult stage was the most tolerant, as it was observed that the larvae died by 100% within the first week of exposure to the dose of 500 Gy, while the death of the adults did not occur until after 3 weeks, when exposed to the dose of 1000 Gy. This was due to Bergonie and Tribondeau’s theory, which noted that tissues appear to be more radiosensitive when the cells are less differentiated and that the cells have greater proliferative capacity and divide more rapidly^34^. The results were in accordance with the findings of Seal and Tilton^37^ who stated that the first, sixth, and seventh instar larvae of *Dermestes maculatus* (DeGeer, 1774) were all killed by an absorbed dose of 200 Gy, whereas the fourth and fifth instar larvae were more resistant. Twenty-four-hour-old pupae treated with 150 Gy failed to eclose, but eclosion was unaffected in older pupae.

Mortality in the larval stage significantly increased with the increasing of gamma radiation doses until it reached a high reduction percentage (90.0 and 100.0%) at doses of 400 and 500 Gy, respectively. Our findings agree with Abdel-Kawy^39^ who reported that larval mortality of the khapra beetle, *Trogoderma granarium* reached 92.5% at a dose of 80 Gy and no larvae survived after exposure to 100 Gy or more. Approximately 69.5, 19.6, and 7.5% of the larvae succeeded in pupating at doses of 40, 60, and 80 Gy, respectively, compared to 94.6% in the control. It was noted from the results that the radiation of pupae led to a delay in the emergence of adult insects and at the highest dose did not develop into adults, indicating the superior performance of the 1000 Gy dose treatment over other treatments. Additionally, the adult stage was more tolerant than the larval and pupal stages, as there was no death during the first week when irradiating the whole adults with all doses except for the high doses of 800 and 1000 Gy. These results confirm the LD_50_ and LD_90_ values, where we found that the highest value when irradiating adults (471.6 and 1027.5 Gy) followed by pupae (298.9 and 922.0 Gy) then larvae (133.9 and 405.5 Gy). These results agree with many works on Coleoptera insects, such as Padwal-Desai et al.^40^ who found no emergence of adults of *Oryzaephilus surinamensis* (Linnaeus, 1758) irradiated with more than 1000 Gy of gamma radiation. Hien^41^ indicated that *D. maculatus* might be completely eliminated at doses as low as 0.1 kGy. Therefore, gamma radiation can be used for the disinfestation of hide beetles to preserve dried fish. Titima^42^ reported that a dose of 1000 Gy completely prevented the emergence of adults in *Lasioderma serricorne* (Fabricius, 1792). Abbas and Nouraddin^43^ reported that a high dose of gamma radiation above 700 Gy controlled population growth and prevented adult emergence of *Tribolium castaneum* (Herbst, 1797). Peter et al.^44^ reported a decrease in the number of adult emergences of *Sitophilus oryzae* (Linnaeus, 1763) with an increase in the dose of gamma radiation. Moreover, the radiation caused deformities in the larvae and pupae, as the hairs scattered on the body of the larva were irregular and their density decreased. Additionally, the highest mortality rate was observed at 800 and 1000 Gy in the pupal stage, where the adult insect's elytra cuticle was not completed, which prevented it from leaving the puparium and eventually caused its death. When *Tribolium confusum* (Jaquelin Du Val, 1868) last instar larvae or early pupae were exposed to radiation, adult emergence was not hindered, but the images showed obvious deformities, as noted by Ducoff and Walburg in^45^. They frequently saw anomalies that resembled split elytra with violent twisting. The severity of the deformity was correlated with the dose administered, and adults with severe deformities had shorter survival times than those with mild deformities. Many authors have reported that radiation directly affects insect behavior, biochemistry, and developmental physiology as it significantly increases oxidative stress^46–48^.

The sterilizing dose is a critical component for the cost-effective application of SIT. This should be kept to a minimum to preserve the insect's behavior. The mode of action of ionizing radiation in living cells consists basically of a chain of oxidative reactions along the radiation path and the formation of free damaging peroxy radicals, which irreversibly alter the organic molecules^49^. At the cytological level, sterilization is the result of germ cell chromosome fragmentation (dominant lethal mutations, translocations and other chromosomal aberrations), leading to the production of imbalanced gametes and subsequently, inhibition of mitosis and the death of fertilized eggs^49^. Irradiation also negatively affected female fecundity and this effect was most severe when irradiated females were crossed with irradiated males. For instance, egg production by irradiated female *A. verbasci* when both sexes were exposed to the same dose (150 Gy) was 26.6 compared to 103.8 eggs in the control. Additionally, this dose completely prevented egg hatching, which indicates that 150 Gy is sufficient to prevent reproduction in this species. Sterility is expressed in terms of egg hatchability in this study. The irradiation dose of 150 Gy caused total sterility without causing acute mortality in the adults. Seal and Tilton^38^ stated that adults of *D. maculatus* that emerged from 72 h old female pupae irradiated with 150 Gy remained infertile, but male pupae needed more than 200 Gy for sterilization. For 24-h-old adults (males and females), a dose of 300 Gy was required to complete sterilization. According to Boshra^50^, every reciprocal mating between nonirradiated and 120 Gy irradiated male and female azuki bean beetles resulted in 100% sterility without impacting survivability. Mansour^35^ also reported that when mixed population of *Trogoderma granarium* Everts (Coleoptera: Dermestidae) was irradiated with 100 Gy, no progeny were produced. Arthur et al.^51^ stated that 125 Gy of gamma radiation was enough to sterilize adults of *Alphitobius diaperinus* (Panzer, 1797) that emerged from irradiated pupae. Additionally, irradiating the pupa with 150 Gy was lethal. Infested peanuts with the *A. diaperinus* pupal phase can therefore be treated phytosanitary with a dose of 150 Gy. Altogether, these reports support our study in that the sterility of gamma- irradiated *A. verbasci* was highly induced, even though there was no acute mortality at the tested doses.

## Conclusion

From this study, we conclude that it is possible to minimize or reduce the damage caused by this dangerous pest, Varied Carpet Beetle, *Anthrenus verbasci,* on antique carpets or textile manufacturers in warehouses or even factories by using the sterilizing dose (150 Gy) or the lethal dose (1000 Gy) of gamma radiation as a safe and environmentally friendly method to eliminate problems caused by using pesticides. These are only preliminary results and additional experiments should be conducted to evaluate the undesirable side effects of high irradiation doses on the physical and chemical structure of organic and inorganic materials. In addition, the recommendation to use ionizing radiation is not sufficient alone as alternative to chemical methods but also as a complement to other control methods.

## Data Availability

All data generated or analyzed during this study are included in this published article.
